# Construct and Validate a Predictive Model for Surgical Site Infection after Posterior Lumbar Interbody Fusion Based on Machine Learning Algorithm

**DOI:** 10.1155/2022/2697841

**Published:** 2022-08-23

**Authors:** Chuang Xiong, Runhan Zhao, Jingtao Xu, Hao Liang, Chao Zhang, Zenghui Zhao, Tianji Huang, Xiaoji Luo

**Affiliations:** ^1^Department of Orthopedics, The First Affiliated Hospital of Chongqing Medical University, Chongqing 400016, China; ^2^Orthopedic Laboratory of Chongqing Medical University, Chongqing, China

## Abstract

**Purpose:**

Surgical site infection is one of the serious complications after lumbar fusion. Early prediction and timely intervention can reduce the harm to patients. The aims of this study were to construct and validate a machine learning model for predicting surgical site infection after posterior lumbar interbody fusion, to screen out the most important risk factors for surgical site infection, and to explore whether synthetic minority oversampling technique could improve the model performance.

**Method:**

This study reviewed 584 patients who underwent posterior lumbar interbody fusion for degenerative lumbar disease at our center from January 2019 to August 2021. Clinical information and laboratory test data were collected from the electronic medical records. The original dataset was divided into training set and validation set in a 1 : 1 ratio. Seven machine learning algorithms were used to develop predictive models; the training set of each model was resampled using synthetic minority oversampling technique. Finally, the model performance was assessed in the validation set.

**Results:**

Of the 584 patients, 33 (5.65%) occurred surgical site infection. Stepwise logistic regression showed that preoperative albumin level (OR 0.659, 95% CI 0.563-0.756), diabetes (OR 9.129, 95% CI 3.816-23.126), intraoperative dural tear (OR 8.436, 95% CI 2.729-25.334), and rheumatic disease (OR 8.471, 95% CI 1.743-39.567) were significant predictors associated with surgical site infection. The performance of the AdaBoost Classification Trees model was the best among the seven machine learning models, and synthetic minority oversampling technique improved the performance of all models.

**Conclusion:**

The prediction model we constructed based on machine learning and synthetic minority oversampling technique can accurately predict surgical site infection, which is conducive to clinical decision-making and optimization of perioperative management.

## 1. Background

Posterior lumbar interbody fusion (PLIF) is a classic operation for the treatment of lumbar degenerative diseases such as lumbar disc herniation, lumbar spinal stenosis, and lumbar spondylolisthesis. Surgical site infection (SSI) is a serious and costly complication, and the reported incidence varies from 0.2% to 16.1% [1]. Surgical site infections can lead to catastrophic consequences such as instrumentation failure, osteomyelitis, pseudoarthrosis, prolonged hospitalization, increased hospital costs, readmissions, and even sepsis or death, increasing patient suffering and placing a heavy burden on families [2]. With the aggravation of the aging population, the number of patients with degenerative lumbar diseases has gradually increased, and correspondingly, the number of those who need to perform this procedure has also increased; at the same time, surgical site infections are increasing; this poses a serious challenge to family and social health systems [3]. Therefore, developing an accurate predictive model for early identification of patients at high risk of surgical site infection and targeted intervention is the most cost-effective approach.

In recent years, artificial intelligence has played an important role in the medical field, such as coronavirus disease 2019 (COVID-19) diagnosis [4], detection of gastrointestinal polyps [5], retinal vessel segmentation [6], image diagnosis of lung cancer [7], diagnosis of atrophic gastritis [8], and confidentiality management of electronic medical records on the cloud [9]. Machine learning, a form of artificial intelligence, combined with medical big data can create algorithms that rival those of human doctors [10]. Unfortunately, few studies have applied machine learning algorithms to predict surgical site infection after posterior lumbar interbody fusion. Therefore, we trained seven machine learning prediction models to early predict the risk of surgical site infection after PLIF using easily available preoperative and intraoperative factors. However, given that most patients after posterior lumbar interbody fusion do not develop surgical site infections, such data structures suffer from category imbalance (which refers to the unequal number of samples between categories in the classification problem), and the effectiveness of machine learning algorithms in this situation is reduced [11]. However, synthetic minority oversampling technique (SMOTE) is a common method to deal with unbalanced data [12]. Therefore, this study uses SMOTE to optimize our machine learning prediction model.

Our study is aimed at developing and validating a machine learning prediction model for surgical site infection after PLIF. To the best of our knowledge, our study is the first to combine SMOTE with multiple machine learning algorithms to develop and validate a predictive model for surgical site infection after PILF. Clinicians can identify high-risk patients with surgical site infection early through this prediction model, which is helpful to optimize patient selection and perioperative management. Early preventive intervention in this population can reduce the occurrence of serious complications and may prevent the occurrence and development of surgical site infection.

## 2. Methods

### 2.1. Patients

This study was approved by the ethics committee of The First Affiliated Hospital of Chongqing Medical University. And the informed consent was waived for the retrospective study. From January 2019 to August 2021, a total of 584 patients underwent posterior lumbar interbody fusion (PLIF) at our center for degenerative lumbar disease. Inclusion criteria were as follows: (1) age ≥ 18 years; (2) diagnosis of lumbar degenerative diseases, including lumbar disc herniation, lumbar spinal stenosis, spondylolisthesis, and lumbar instability based on lumbar magnetic resonance imaging (MRI) and clinical manifestations; (3) undergoing primary single-level or multilevel PLIF surgery. The exclusion criteria were as follows: (1) patients with a previous history of open lumbar surgery; (2) patients with preoperative concurrent active infection of the spine or other parts of the body, spinal deformity, and tumors.

All operations and perioperative management were performed by the same experienced spine surgical team. All procedures were performed in a standard vertical stratospheric operating room. We performed antibiotic prophylaxis 30 minutes before the start of surgery and extended it to 72 hours after surgery, and uniform criteria were adopted for the type, time, and dose of perioperative antibiotics. All patients were asked to follow the same wound care and functional exercise protocol.

SSI was defined according to the Centers for Disease Control (CDC) and prevention criteria [13, 14]. Patients who meet one of the following conditions can be diagnosed with SSI (monitored for 90 days after surgery): (1) clinical manifestations such as redness, swelling, heat, pain, tenderness, and/or purulent drainage appear in the wound; (2) the abscess was aspirated from the wound surface, and the culture was positive; (3) positive fluid or tissue culture collected during revision surgery; (4) histopathological and radiological examination confirmed SSI evidence; (5) SSI is diagnosed by the surgeon and clearly recorded in the medical record. SSI can be divided into superficial infection and deep infection according to the location of occurrence.

### 2.2. Data Collection

The following clinical information of the patients was retrospectively collected through the electronic medical record system, surgical anesthesia system, and mobile nursing system: the clinical information of the patients, including age, the American Society of Anesthesiologists (ASA) classification, New York Heart Association (NYHA) classification, body mass index (BMI), smoking, drinking, whether there was a history of rheumatic disease, whether there was osteoporosis, hypertension, diabetes, diagnosis, and whether it was cold season or warm season at discharge. Routine laboratory tests, including routine blood tests, liver function tests, and renal function tests, were collected. At the same time, we recorded surgery-related parameters, including operation time, estimated intraoperative blood loss, number of fusions, and whether there was dural tear during the operation. Smoking status was classified as current smoking, regardless of the amount or type of tobacco smoked, and all passive smokers and former smokers were considered nonsmokers. The American Society of Anesthesiologists physical status is a classification that evaluates a patient's physical status before surgery [15]; it is also commonly used in preoperative risk prediction in recent years [16], coded according to the 1963 American Society of Anesthesiologists five-level classification system of physical conditions (1 = a healthy individual, 2 = mild systemic disease, 3 = severe systemic disease, 4 = persistent life-threatening severe systemic disease, and 5 = a dying person who is not expected to survive with or without operation). Patients with rheumatoid arthritis, ankylosing spondylitis, psoriatic arthritis, or systemic lupus erythematosus were considered to have a history of rheumatic diseases.

### 2.3. Synthetic Minority Oversampling Technique [12]

We divided the original dataset into training set and verification set according to 1 : 1 and resampled the training set of each model using the synthetic minority oversampling technique. It should be pointed out that we did not resample the validation set.

### 2.4. Development and Validation of Machine Learning Models

In this study, univariate logistic regression and multivariate logistic regression were used, and then, factors that were significant in both univariate and multivariate analyses were included in stepwise logistic regression to determine the important factors associated with SSI after PLIF. Next, the dataset was randomly divided into training set and validation set, each accounting for 50% of the study cohort. In order to solve the problem of data imbalance, SMOTE algorithm was used to preprocess the training set. Machine learning algorithms (Boosted Classification Trees [17], Boosted Logistic Regression [18], Extreme Gradient Boosting [19], Stochastic Gradient Boosting [20], Generalized Linear Model [21], AdaBoost Classification Trees [22], and Random Forest [23]) model the training set, then the accuracy of the model was verified in the validation set.

### 2.5. Model Evaluation

To evaluate the performance of the machine learning model, the confusion matrix, accuracy, precision, recall, *F*1 score, *F*3 score, and the area under the receiver-operating characteristic (AUC) value of the machine learning model were calculated in the validation set. Among them, accuracy, precision, recall, and *Fα* score determined by the following formula: in these formulas, TP: true positive; TN: true negative; FP: false positive; FN: false negative. Confusion matrix is a form of summarizing prediction results of classification prediction model in machine learning. The rows of confusion matrix represent predicted values, and the columns of the matrix represent true values. *Fα* score is the result of comprehensive consideration of precision and recall, indicating that the weight of recall is *α* times of precision weight in the scoring generation process. *Fα* score was, respectively, calculated when *α* is 1, 2, and 3, and *F*3 was finally determined as the evaluation index of the model. A good model should have high *Fα* scores and AUC values when evaluating the performance of different machine learning algorithms. Compare the predictive performance of the seven machine learning models before and after preprocessing training sets using synthetic minority oversampling technique. The algorithm with the best performance was taken as the final prediction model, and the importance of variables was ranked.

(1) $$\begin{array}{c} \text{Accuracy}=\frac{\text{TP}+\text{TN}}{\text{TP}+\text{TN}+\text{FP}+\text{FN}},\\ \text{Precision}=\frac{\text{TP}}{\text{TP}+\text{FP}},\\ \text{Recall}=\frac{\text{TP}}{\text{TP}+\text{FN}},\\ F\alpha \text{ score}=\frac{\left(1+\alpha^{2}\right)*\text{Precision}*\text{Recall}}{\left(\alpha^{2}*\text{Precision}\right)+\text{Recall}}. \end{array}$$

### 2.6. Statistical Analysis

R software was used for statistical analysis, using caret package, DMwR package, NNET package, random forest package, RSNNS package, klaR package, fast Adaboost package, and forest plot package. For continuous and normally distributed variables, *t*-test of two independent samples is used to analyze and compare. Otherwise, Mann–Whitney *U*-test was used for comparison between groups. Qualitative variables were analyzed by chi-square test. *P* value < 0.05 was considered statistically significant.

## 3. Results

### 3.1. Patients' Characteristics

After screening for inclusion and exclusion criteria, a total of 584 consecutive patients (54.97% female, 45.03% male) who underwent posterior lumbar fusion for degenerative lumbar disease were included in the study, and 33 patients (5.65%) with surgical site infection. There were significant differences in age (*P* < 0.001), preoperative red blood cell count (*P* = 0.041), preoperative albumin level (*P* < 0.001), number of surgical fusion segments (*P* < 0.001), intraoperative dural tears (*P* < 0.001), presence of diabetes (<0.001), history of rheumatic disease (*P* = 0.002), ASA grade (*P* = 0.01) between the infection group and the noninfection group, but there were no significant differences in other variables between the two groups (Table 1).

### 3.2. Logistic Regression

**Figure 1:**
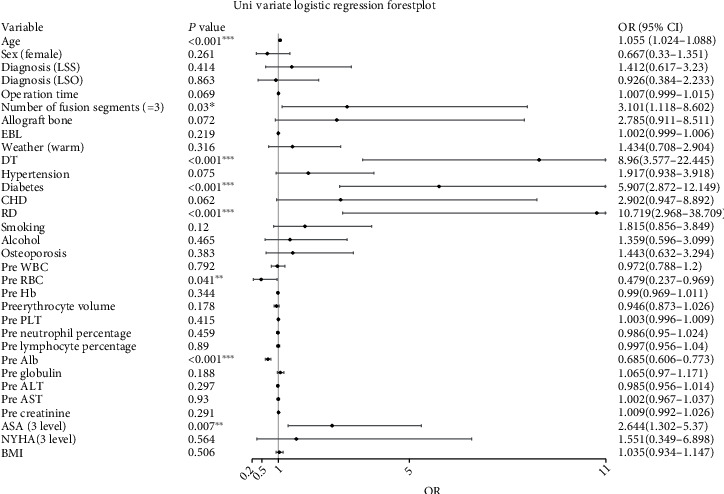
Univariate logistic regression. LSS: lumbar spinal stenosis; LSO: lumbar spondylolisthesis; EBL: estimated blood loss; DT: dural tear; RD: rheumatic disease; CHD: coronary heart disease; Pre WBC: preoperative white blood cell count; Pre RBC: preoperative red blood cell count; Pre Hb: preoperative hemoglobin; Pre erythrocyte volume: preoperative erythrocyte volume; Pre PLT: preoperative platelets; Pre neutrophil percentage: preoperative neutrophil percentage; Pre lymphocyte percentage: preoperative lymphocyte percentage; Pre Alb: preoperative albumin; Pre globulin: preoperative globulin; Pre ALT: preoperative alanine aminotransferase; Pre AST: preoperative aspartate aminotransferase; Pre creatinine: preoperative creatinine; ASA: American Society of Anesthesiologists physical status; NYHA: New York Heart Association Class; BMI: body mass index. ^∗^*P* value < 0.05; ^∗∗^*P* value < 0.01; ^∗∗∗^*P* value < 0.001.

**Figure 2:**
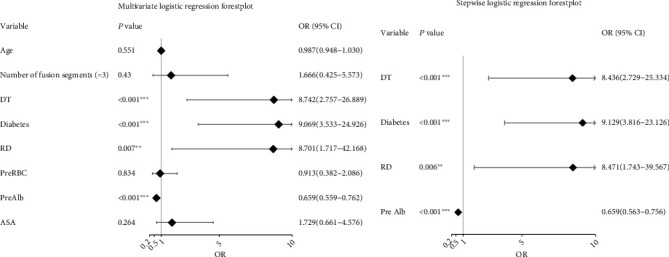
Multivariate logistic regression and stepwise logistic regression. DT: dural tear; RD: rheumatic disease; Pre RBC: preoperative red blood cell count; Pre Alb: preoperative albumin; ASA: American Society of Anesthesiologists physical status. ^∗^*P* value < 0.05; ^∗∗^*P* value < 0.01; ^∗∗∗^*P* value < 0.001.

Univariate analysis showed that the factors with statistical significance (*P* < 0.05) were age, number of fusion levels, intraoperative dural tear, diabetes, history of rheumatic disease, preoperative red blood cell count, preoperative albumin level, and ASA grade (Figure 1). Multivariate analysis showed that factors with statistical significance (*P* < 0.05) included intraoperative dural tear, diabetes, history of rheumatic disease, and preoperative albumin level (Figure 2(a)). Stepwise logistic regression identified the best variables for inclusion in the machine learning model, including intraoperative dural tear (OR 8.436, 95% CI 2.729-25.334), diabetes (OR 9.129 3.816-23.126), history of rheumatic disease (OR 8.471, 1.743-39.567), and preoperative albumin level (OR 0.659 0.563-0.756) (Figure 2(b)).

### 3.3. Machine Learning Predictive Model Performance

**Figure 3:**
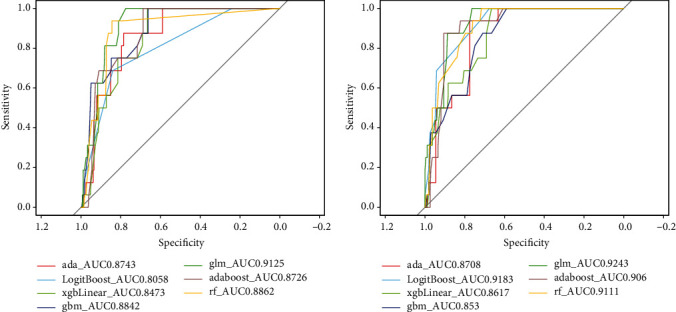
Area under the curve of receiver-operating characteristic curve by machine learning models in the validation cohort. (a) Machine learning; (b) SMOTE + machine learning. AUC: area under the receiver-operating characteristic curve; SMOTE: synthetic minority oversampling technique; ada: Boosted Classification Trees; LogitBoost: Boosted Logistic Regression; xgbLinear: Extreme Gradient Boosting; gbm: Stochastic Gradient Boosting; glm: Generalized Linear Model; adaboost: AdaBoost Classification Trees; rf: random forest.

**Figure 4:**
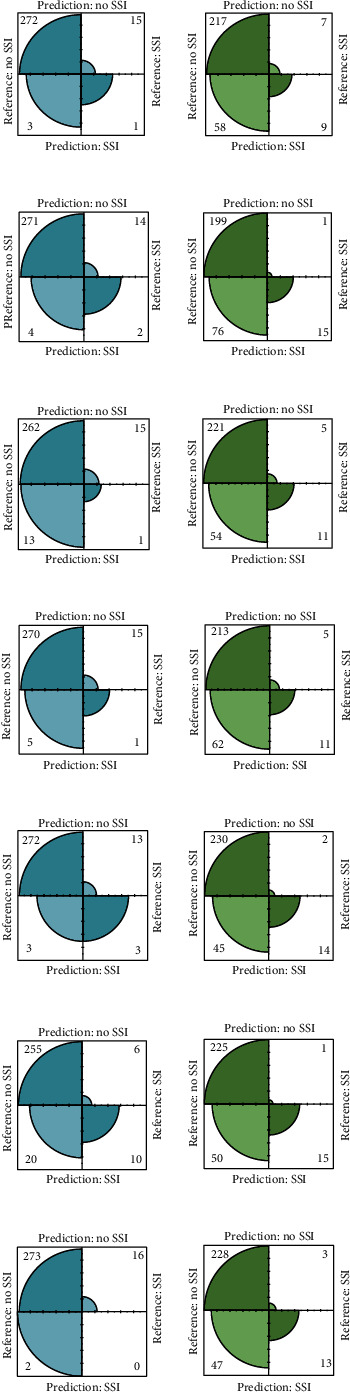
Confusion matrix of prediction model for validation set. SSI: surgical site infection; no SSI: no surgical site infection; (a) Boosted Classification Trees; (b) SMOTE+Boosted Classification Trees; (c) Boosted Logistic Regression; (d) SMOTE+Boosted Logistic Regression; (e) Extreme Gradient Boosting; (f) SMOTE+Extreme Gradient Boosting; (g) Stochastic Gradient Boosting; (h) SMOTE+Stochastic Gradient Boosting; (i) Generalized Linear Model, (j) SMOTE+Generalized Linear Model; (k) AdaBoost Classification Trees; (l) SMOTE+AdaBoost Classification Trees; (m) random forest; (n) SMOTE+Random Forest.

**Figure 5:**
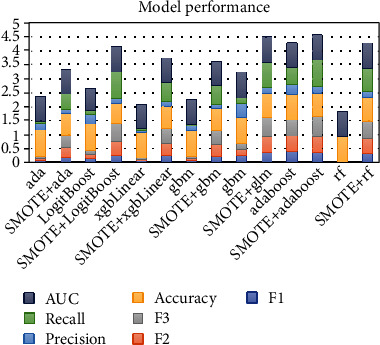
Model performance. AUC: area under the receiver-operating characteristic curve; SMOTE: Synthetic Minority Oversampling Technique; ada: Boosted Classification Trees; LogitBoost: Boosted Logistic Regression; xgbLinear: Extreme Gradient Boosting; gbm: Stochastic Gradient Boosting; glm: Generalized Linear Model; adaboost: AdaBoost Classification Trees; rf: random forest.

The comparison of the prediction performance of the seven machine learning models in the validation set is shown in Figures 3–5. AdaBoost Classification Trees model has the best performance, with AUC of 0.8726, recall of 0.6250, precision of 0.3333, accuracy of 0.9107, and *F*3 of 0.5747 for the model without synthetic minority oversampling technique. The confusion matrix shows that only the AdaBoost Classification Trees model can accurately identify the patients with high risk of infection in the validation set (correctly identify 10 of the 16 high-risk patients in the validation set) before the combination of synthetic minority oversampling technique. Although other models had high accuracy and AUC values, they failed to identify patients at high risk of surgical site infection. The AdaBoost Classification Trees model combined with synthetic minority oversampling technique showed better prediction performance, which correctly identified 15 of the 16 high-risk patients of infection in the validation set. Its AUC was 0.906, recall was 0.9375, precision was 0.2308, accuracy was 0.8247, and *F*3 was 0.7177. The prediction performance of the remaining models for patients at high risk of infection was also significantly improved by synthetic minority oversampling technique, as shown in Figure 4.

### 3.4. Variable Importance

**Figure 6:**
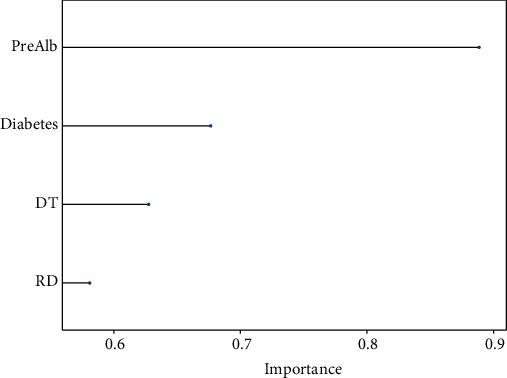
Variable importance. Pre Alb: preoperative albumin; DT: dural tear; RD: rheumatic disease.

In the AdaBoost Classification Trees model, the relative importance of variables is shown in Figure 6, in descending order of importance as follows: preoperative albumin level, diabetes, intraoperative dural tear, and history of rheumatic disease.

## 4. Discussion

In this study, we developed and validated a predictive model for surgical site infection after posterior lumbar interbody fusion using multiple machine learning algorithms and SMOTE. We found that SMOTE used in the training set improved the performance of the prediction model, and the AdaBoost Classification Trees model combined with SMOTE provided the best performance compared to other models. This predictive model based on SMOTE and machine learning can help early identify patients at high risk for surgical site infection, optimize perioperative management, and facilitate clinical decision-making.

Surgical site infection has always been a concern for spinal surgeons; the surgical site infection rate after posterior lumbar interbody fusion was 5.65%, which was consistent with previous studies [1]. Although studies have reported risk factors for surgical site infection after lumbar interbody fusion [24], however, these studies only described risk factors as relative risk (RR) or odds ratio (OR), which is not sufficient to comprehensively assess the risk of surgical site infection after PLIF for individual patients. Therefore, we used machine learning algorithms to develop a predictive model for surgical site infection after posterior lumbar interbody fusion, which is the first prediction model to predict surgical site infection after PILF using synthetic minority oversampling techniques and machine learning algorithms in imbalanced datasets.

Synthetic minority oversampling technique is an algorithm that combines oversampling of minority classes with undersampling of majority classes. It is a common method to deal with data imbalance. It can construct new minority samples rather than directly copy the minority samples, that is, the data constructed by the algorithm is new samples and does not exist in the original dataset [25]. It selects two or more similar samples under the small category based on the distance measure, then selects one of the samples, and randomly selects a certain number of adjacent samples to add noise to an attribute of the selected sample, so as to construct more new data [12].

Unbalanced data refers to the unequal number of samples between categories in classification problems [25]. In our study, patients with SSI accounted for 5.65%, while patients without SSI accounted for 94.35%. Therefore, data imbalance existed in this study. When dealing with the classification problem of imbalanced data, machine learning prediction models tend to predict all results into most classes to achieve high accuracy [26]. However, when minority categories are more important (in this case, identifying patients at high risk for surgical site infection is more important), imbalanced data often leads to poor predictive performance. The synthetic minority oversampling technique and ensemble learning method are commonly used to deal with data imbalance [27]. Therefore, we used SMOTE to oversampling the minority (abnormal) classes and undersampling the majority (normal) classes in the training set to overcome this problem (class imbalance) and optimize our machine learning algorithm [12]. Previous studies have also shown that synthetic minority oversampling technique helps improve model accuracy without compromising research results [26, 28]. Our study confirmed that applying synthetic minority oversampling technique to the training set can improve the performance of machine learning prediction models when we need to improve the sensitivity of the model without losing too much specificity.

Since the purpose of the prediction model proposed in this study is to identify patients at risk for surgical site infection, the sensitivity of the model is more important than the specificity. In this study, the AdaBoost Classification Trees model combined with synthetic minority oversampling technique successfully predicted 15 patients at high risk of infection in the validation set, but at the cost of misidentifying 50 (17%) patients at low risk of infection as high risk. For patients predicted to be at high risk of infection, clinicians can apply more stringent glycemic control, close monitoring of albumin levels, and more rigorous wound care. If patients have multiple high-risk risk factors, clinicians may adjust the type and duration of antibiotic use as appropriate. These measures are an acceptable burden for patients predicted to be at high risk of infection, while reducing the rate of missed diagnosis and reducing the incidence of SSI in the overall population.

*Fα* score is a model evaluation index that comprehensively considers precision and recall, indicating that the weight of recall is *α* times of precision when generating scores. *α* < 1 indicates that the precision of the model is more important. *A* > 1 indicates that the recall of the model is more important. In clinical practice, early identification and stratification of patients at high risk of infection may be beneficial for better prevention of surgical site infection. We did not want to miss any patients at high risk of postoperative infection, that is, we wanted to emphasize the recall rate of the model over the precision. We calculated *Fα* scores when *α* values were 1, 2, and 3, respectively, and finally determined *F*3 as the most important index to evaluate the model performance. The results of this study also prove that it is necessary to appropriately expand the *α* value in the study of severe and infrequent complications.

Our study further confirms that low preoperative albumin levels, diabetes, history of rheumatic disease, and intraoperative dural tear are risk factors for surgical site infection after posterior lumbar interbody fusion, which is consistent with previous findings [24, 29]. Therefore, preoperative optimization of nutritional status, perioperative monitoring of albumin, and careful intraoperative operation to avoid dural injury may help prevent surgical site infection.

To the best of our knowledge, this study is the first to use SMOTE combined with machine learning algorithms to develop and validate a predictive model for surgical site infection after PILF. Clinicians can use this prediction model to preliminarily identify the high-risk population for SSI and conduct early preventive intervention to reduce the incidence of serious complications. Examples include correction of hypoproteinemia and careful intraoperative procedures to avoid dural tears. In addition, since most lumbar degenerative diseases are elective surgeries, clinicians can preliminarily judge the risk of surgical site infection through the prediction model proposed in this study, grasp the timing of surgery, weigh the advantages and disadvantages of surgery, and answer the consultation of patients about infection complications. The synthetic minority oversampling technique is an effective method to improve the prediction performance of machine learning prediction models for unbalanced datasets. Similar imbalanced data exist for many diseases and postoperative complications [30]; synthetic minority oversampling technique used in this study can be applied to study of other diseases. Synthetic minority oversampling technique may be a feasible method to improve the performance of machine learning prediction model as a data pretreatment process.

## 5. Conclusion

Our prediction model based on machine learning and SMOTE can successfully predict patients at high risk of infection. It is helpful for clinicians to optimize patient selection and timing of surgery (such as elective surgery after correcting hypoalbuminemia in high-risk patients) and answer patients' consultation on infection complications; early identification and early intervention can reduce the occurrence of serious complications and may prevent the occurrence of surgical site infections. The method adopted in this study also provides reference for the study of other diseases and complications. The limitation of this study is that the single-center retrospective study may introduce selection bias and limit its generalization, which needs to be verified in more and broader populations in the future. Meanwhile, in addition to the methods used in this study, we are looking forward to more future research using some of the most representative computational intelligence algorithms which can be used to solve the problems, like monarch butterfly optimization (MBO) [31], earthworm optimization algorithm (EWA) [32], elephant herding optimization (EHO) [33], moth search (MS) algorithm [34], slime mould algorithm (SMA) [35], hunger games search (HGS) [36], Runge Kutta optimizer (RUN) [37], colony predation algorithm (CPA) [38], and Harris hawks optimization (HHO) [39].

## Figures and Tables

**Table 1:** Baseline characteristics of all patients included in the study.

	Total	Non-SSI	SSI	*P* value
Number of patients	584	551 (94.35%)	33 (5.65%)	
Age (years)	58.36 ± 13.76	57.86 ± 13.86	66.70 ± 8.60	<0.001
Operation time	145.56 ± 40.48	144.81 ± 40.06	158.06 ± 45.77	0.095
EBL	151.23 ± 81.04	150.22 ± 80.95	168.18 ± 81.79	0.125
Pre WBC	5.94 ± 1.71	5.94 ± 1.72	5.86 ± 1.50	0.937
Pre RBC	4.44 ± 0.55	4.45 ± 0.54	4.25 ± 0.59	0.041
Pre Hb	134.76 ± 16.33	134.92 ± 16.30	132.15 ± 16.96	0.229
Pre erythrocyte volume	40.94 ± 4.38	41.00 ± 4.36	39.94 ± 4.67	0.179
Pre PLT	205.67 ± 55.58	205.21 ± 54.98	213.33 ± 65.29	0.619
Pre neutrophil percentage	58.03 ± 9.47	58.10 ± 9.56	56.84 ± 7.98	0.490
Pre lymphocyte percentage	31.09 ± 8.42	31.10 ± 8.50	30.89 ± 6.96	0.795
Pre Alb	42.56 ± 3.47	42.80 ± 3.34	38.64 ± 3.24	<0.001
Pre globulin	24.34 ± 3.65	24.29 ± 3.66	25.15 ± 3.29	0.137
Pre ALT	22.47 ± 17.56	22.66 ± 17.83	19.39 ± 12.02	0.658
Pre AST	20.40 ± 9.82	20.39 ± 9.96	20.55 ± 7.34	0.342
Pre creatinine	68.66 ± 17.69	68.47 ± 17.65	71.82 ± 18.19	0.208
BMI	24.22 ± 3.33	24.20 ± 3.34	24.59 ± 3.16	0.456
Sex (%)				0.342
Female	321 (54.97)	306 (55.54)	15 (45.45)	
Male	263 (45.03)	245 (44.46)	18 (54.55)	
Diagnosis (%)				0.625
Lumbar disc herniation	284 (48.63)	269 (48.82)	15 (45.45)	
Lumbar spinal stenosis	137 (23.46)	127 (23.05)	10 (30.3)	
Lumbar instability/spondylolisthesis	163 (27.91)	155 (28.13)	8 (24.24)	
Number of fusion segments				0.040
1-2	549 (94)	521 (94.56)	28 (84.8)	
≥3	35 (6)	30 (5.44)	5 (15.2)	
Allograft bone				0.081
No	554 (94.86)	525 (95.28)	29 (87.9)	
Yes	30 (5.14)	26 (4.72)	4 (12.1)	
Weather				0.408
Cold	315 (53.94)	300 (54.45)	15 (45.45)	
Warm	269 (46.06)	251 (45.55)	18 (54.55)	
Dural tear				<0.001
No	557 (95.38)	532 (96.55)	25 (75.76)	
Yes	27 (4.62)	19 (3.45)	8 (24.24)	
Hypertension				0.107
No	417 (71.4)	398 (72.23)	19 (57.58)	
Yes	167 (28.6)	153 (27.77)	14 (42.42)	
Diabetes				<0.001
No	483 (82.71)	467 (84.75)	16 (48.48)	
Yes	101 (17.29)	84 (15.25)	17 (51.52)	
CHD				0.073
No	555 (95.03)	526 (95.46)	29 (87.88)	
Yes	29 (4.97)	25 (4.54)	4 (12.12)	
RD				0.002
No	573 (98.12)	544 (98.73)	29 (87.88)	
Yes	11 (1.9)	7 (1.27)	4 (12.12)	
Smoking				0.174
No	454 (77.74)	432 (78.40)	22 (66.67)	
Yes	130 (22.26)	119 (21.60)	11 (33.33)	
Alcohol				0.613
No	471 (80.65)	446 (80.94)	25 (75.76)	
Yes	113 (19.35)	105 (19.06)	8 (24.24)	
Osteoporosis				0.519
No	476 (81.51)	451 (81.85)	25 (75.76)	
Yes	108 (18.49)	100 (18.15)	8 (24.24)	
ASA				0.010
1-2	394 (67.47)	379 (68.78)	15 (45.45)	
3	190 (32.53)	172 (31.22)	18 (54.55)	
NYHA				0.639
≤2	560 (95.89)	529 (96.01)	31 (93.94)	
3	24 (4.11)	22 (3.99)	2 (6.06)	

EBL: estimated intraoperative blood loss; Pre WBC: preoperative white blood cell count; Pre RBC: preoperative red blood cell count; Pre Hb: preoperative hemoglobin; Pre erythrocyte volume: preoperative erythrocyte volume; Pre PLT: preoperative platelets; Pre neutrophil percentage: preoperative neutrophil percentage; Pre lymphocyte percentage: preoperative lymphocyte percentage; Pre Alb: preoperative albumin; Pre globulin: preoperative globulin; Pre ALT: preoperative alanine aminotransferase; Pre AST: preoperative aspartate aminotransferase; Pre creatinine: preoperative creatinine; BMI: body mass index; CHD: coronary heart disease; ASA: American Society of Anesthesiologists; NYHA: New York Heart Association Class; SSI: surgical site infection; RD: rheumatic disease.

## Data Availability

The datasets generated and/or analyzed during the current study are not publicly available due to the data is confidential patient data but are available from the corresponding author on reasonable request.
